# A Comprehensive Review of Metaheuristic Algorithms for Node Placement in UAV Communication Networks

**DOI:** 10.3390/s26030869

**Published:** 2026-01-28

**Authors:** S. A. Temesheva, D. A. Turlykozhayeva, S. N. Akhtanov, N. M. Ussipov, A. A. Zhunuskanov, Wenbin Sun, Qian Xu, Mingliang Tao

**Affiliations:** 1 Nonlinear Information Processes Laboratory (NIPL), Faculty of Physics and Technology, Al-Farabi Kazakh National University, Almaty 050040, Kazakhstan; temesheva_symbat4@live.kaznu.kz (S.A.T.); nurzhan.ussipov@kaznu.edu.kz (N.M.U.); zhunuskanov_alisher@live.kaznu.kz (A.A.Z.); 2 Information and Communication Engineering Faculty, Northwestern Polytechnical University, Xi’an 710072, China

**Keywords:** metaheuristic algorithms, UAV communication networks, node placement, coverage, connectivity

## Abstract

Unmanned Aerial Vehicle Communication Networks (UAVCNs) have emerged as a transformative solution to enable resilient, scalable, and infrastructure-independent wireless communication in urban and remote environments. A key challenge in UAVCNs is the optimal placement of Unmanned Aerial Vehicle (UAV) nodes to maximize coverage, connectivity, and overall network performance while minimizing latency, energy consumption, and packet loss. As this node placement problem is NP-hard, numerous meta-heuristic algorithms (MHAs) have been proposed to find near-optimal solutions efficiently. Although research in this area has produced a wide range of meta-heuristic algorithmic solutions, most existing review articles focus on MANETs with terrestrial nodes, while comprehensive reviews dedicated to node placement in UAV communication networks are relatively scarce. This article presents a critical and comprehensive review of meta-heuristic algorithms for UAVCN node placement. Beyond surveying existing methods, it systematically analyzes algorithmic strengths, vulnerabilities, and future research directions, offering actionable insights for selecting effective strategies in diverse UAVCN deployment scenarios. To demonstrate practical applicability, selected hybrid algorithms are evaluated in a reproducible Python framework using computational time and coverage metrics, highlighting their ability to optimize multiple objectives and providing guidance for future UAVCN optimization studies.

## 1. Introduction

In recent years, the rapid advancement of UAVs has driven innovations in communication networks, leading to adaptive architectures tailored to UAV operations [1,2]. Among these, UAV Communication Networks (UAVCNs) have emerged as a transformative solution, enabling reliable, flexible, and infrastructure-independent communication between aerial nodes. A key development in this field is Flying Ad Hoc Networks (FANETs), a specialized extension of Mobile Ad Hoc Networks (MANETs), where UAVs establish wireless links without relying on fixed infrastructure, enabling dynamic communication in remote or disaster-affected areas [3,4].

Expanding this concept, UAV-Assisted Cellular Networks and UAV-based relay networks use UAVs as intermediate nodes to forward data, ensuring resilient and adaptable connectivity [5]. UAVs can act as flying base stations, dynamically adjusting positions based on user density or network demands to optimize coverage, capacity, and reliability, especially in areas with limited infrastructure or during emergencies [6,7]. Similarly, UAV–satellite integrated networks enhance connectivity in both urban and remote environments.

Integration of UAV-based Mobile Edge Computing (MEC) further reduces latency and improves responsiveness [8], while UAV swarms support large-scale cooperative missions and persistent surveillance [9]. Additionally, combining UAVs with massive MIMO technology enhances spectral efficiency, wireless coverage, and connectivity, playing a key role in next-generation 5G and emerging 6G networks [10,11].

Although UAVCNs have been widely adopted, the UAV node placement problem, known to be NP-hard, remains unsolved optimally. Determining the optimal positions of UAVs to maximize coverage and reliability while minimizing latency and packet loss is a combinatorial problem, for which exhaustive search is computationally infeasible due to the exponential growth of possible configurations. Further complicating this challenge is the diversity of objectives and performance metrics used across studies, including coverage, throughput, energy efficiency, network lifetime, and latency, making direct comparison of meta-heuristic algorithms difficult. In addition, real-world scenarios involve dynamic UAV mobility, energy constraints, obstacles, and environmental factors such as weather, while most meta-heuristic approaches are tested on simplified models or simulations, limiting their practical applicability [12,13].

To address these challenges, numerous meta-heuristic algorithms (MHAs) have been proposed for UAVCN node placement, offering advantages such as computational efficiency and ease of implementation, which make them suitable for practical scenarios [14]. However, most existing review studies focus on MANETs or terrestrial networks, and very few address UAVCN node placement; these are not comprehensive or detailed.

In the scientific literature, a range of studies [15,16,17,18,19,20,21,22] address the UAV node placement problem using meta-heuristic algorithms and report comparative evaluations based on performance metrics such as coverage, connectivity, energy consumption, throughput, delay and packet delivery ratio. These works primarily focus on proposing novel or improved meta-heuristic algorithms and validating their effectiveness through comparisons with existing methods in specific scenarios, including smart cities, FANET formation, post-disaster communications, and UAV-based base station deployment. Although they provide experimental results and valuable performance insights, these studies are not presented as review or survey articles and do not aim to provide a systematic, structured and comprehensive analysis of meta-heuristic approaches for UAVCN node placement.

This article presents a comprehensive review of meta-heuristic algorithms applied to the UAVCN node placement problem. It establishes the theoretical foundations of the features of the UAVCN network, details the problem of node placement and its objectives, presents major algorithms’ families, categorizes approaches by principles, and systematically analyzes the strengths, limitations, and potential future directions of each class. By summarizing the key features of the algorithms presented in the discussed articles, along with evaluated metrics and platforms, and by integrating a critical discussion, this article provides both a systematic overview and actionable insights, enabling researchers to understand algorithmic trade-offs and select appropriate strategies for different UAVCN deployment scenarios. To further illustrate practical performance, selected hybrid algorithms are evaluated in a reproducible Python 3.13.2 framework using computational time and coverage metrics, demonstrating their potential to optimize multiple objectives in controlled experimental scenarios.

This article is structured as follows: Section 2 discusses the definition, architecture, and applications of different key subcategories of UAVCNs. Section 3 defines the formulation of the node placement problem and the placement objectives. Section 4 discusses various MHAs, along with illustrative flowcharts that depict their operational workflows. Section 5 presents a discussion of meta-heuristic algorithms for UAV placement, touching upon open problems and future challenges.

## 2. UAVCN

**Figure 1:**
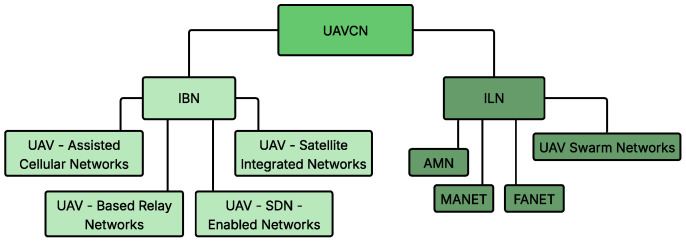
The classification of UAVCN.

UAVCNs integrate UAVs with wireless networking technologies, providing efficient and scalable communication solutions for various applications. Wireless networks are categorized into two main domains: infrastructure-based networks (IBN) and infrastructure-less networks (ILN) [23,24], as illustrated in Figure 1.

**Figure 2:**
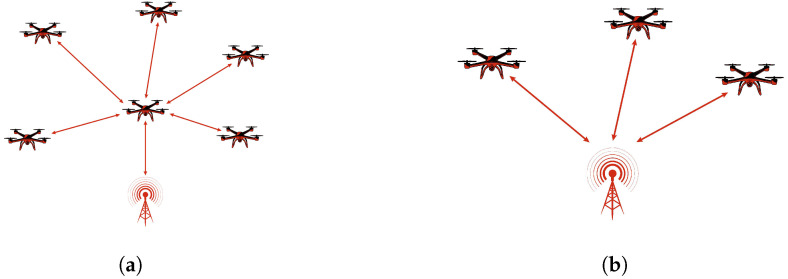
UAVCN architectures. (**a**) ILN architecture. (**b**) IBN architecture.

The ILN encompasses MANET, FANET, Aerial Mesh Networks (AMNs), and UAV Swarm Networks. The IBN consists of UAV-Assisted Cellular Networks, UAV-Based Relay Networks, UAV-Satellite Integrated Networks, and UAV-SDN-Enabled Networks. Figure 2 presents the network architectures of ILN and IBN.

### 2.1. ILN

Infrastructure-less networks provide a flexible and efficient communication framework, particularly in scenarios where infrastructure-based networks are impractical or infeasible. Figure 2a illustrates a typical ILN architecture in which the nodes are fully self-organized and configured, communicating without base stations or access points. Unlike traditional networks, infrastructure-less networks adapt to dynamic topologies, where nodes establish direct wireless links and frequently change positions. This adaptability makes them ideal for applications requiring rapid and temporary network deployment, such as disaster recovery, military operations, and border security. A key advantage of these networks is their ability to support heterogeneous nodes within a defined coverage area, ensuring seamless communication through single-hop and multi-hop transmission. One of the most common examples of infrastructure-less networks includes MANETs, FANETs and AMNs, where nodes dynamically establish connections and communicate using specialized routing protocols without relying on fixed infrastructure. These networks enhance reliability, scalability, and communication efficiency, making them essential for various real-time and mission-critical applications [25].

#### 2.1.1. MANET

**Figure 3:**
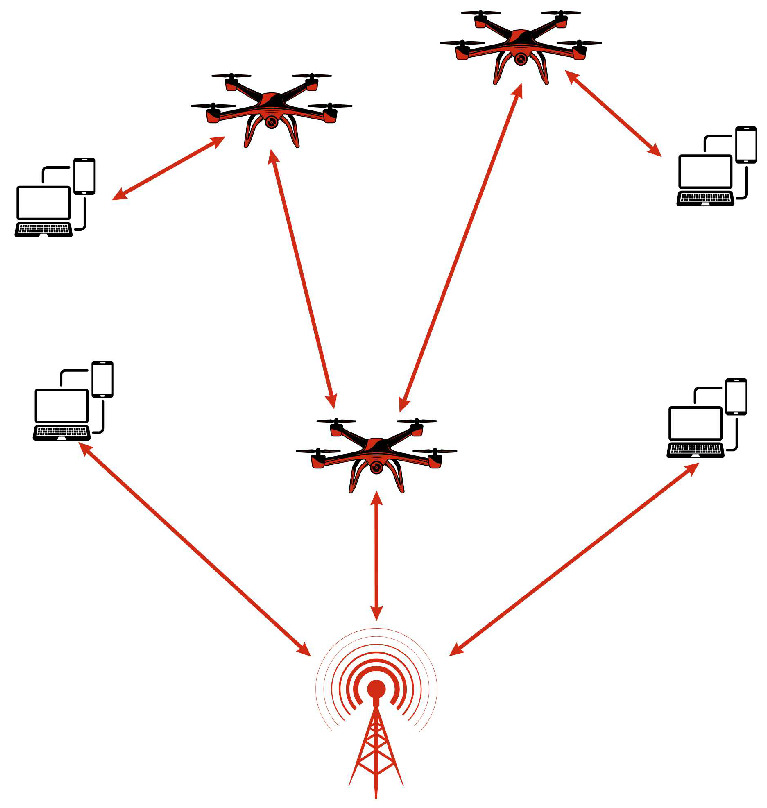
MANET architecture.

A MANET is a dynamic and self-configuring wireless network composed of independent mobile nodes that act as both hosts and routers. MANETs are widely used in military deployments, disaster management, and vehicular communication, where rapid and flexible deployment is essential. Originally, MANETs use wireless links such as IEEE 802.11 [26] and 802.16 [27], allowing nodes like smartphones, notebooks and tablets to establish adaptive networks. The increasing adoption of MANETs has led to their integration with UAV-based networks, where drones serve as mobile nodes to enhance connectivity in challenging environments. In MANETs, drones dynamically adjust their positions to maintain stable communication links, enabling real-time data exchange between relief teams, command centers, and IoT devices. These networks play a crucial role in emergency scenarios by providing vital communication infrastructure in the absence of traditional networks [26,27]. Figure 3 illustrates the architecture of MANET.

#### 2.1.2. FANET

**Figure 4:**
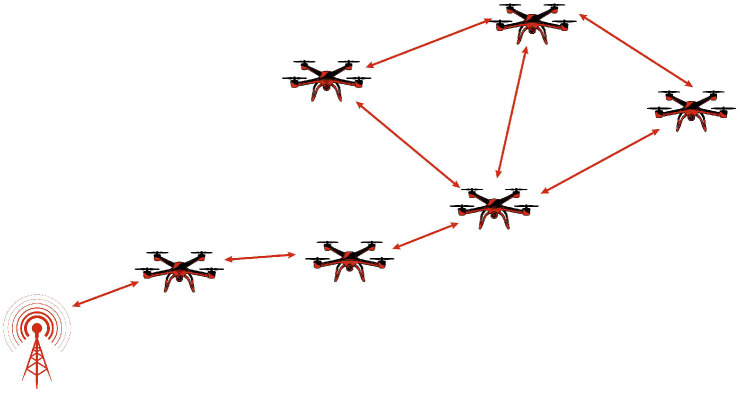
FANET architecture.

FANETs are a distinct category of MANETs specifically designed to enable efficient communication among UAVs. Unlike traditional MANETs, which primarily consist of ground-based mobile devices like smartphones and laptops, FANETs operate in highly dynamic airborne environments, where rapid node mobility, frequent topology changes, and extended communication ranges introduce unique networking challenges. Additionally, FANETs consider variations in altitude, extended communication distances, and intermittent link reliability, aspects that are less prominent in conventional MANETs. A key feature of FANETs is their dual role in both UAV-to-UAV communication and data acquisition, where UAVs collect and transmit environmental information to ground control stations. To ensure reliable connectivity, FANETs utilize high-gain directional antennas, multi-hop relays, and long-range transmission technologies, enabling efficient and scalable aerial networking [28,29]. Figure 4 illustrates the architecture of FANET.

#### 2.1.3. AMN

**Figure 5:**
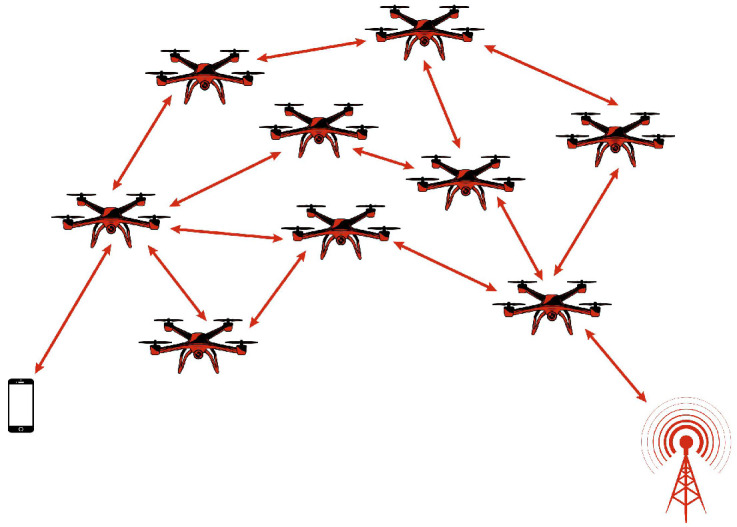
AMN architecture.

AMNs are a specialized form of FANETs consisting of interconnected aerial nodes, including UAVs, balloons, aerostats, and piloted aircraft. Among these, UAVs are the predominant choice due to their cost effectiveness, ease of deployment, and high mobility. AMNs leverage an ad hoc-based mesh topology, enabling seamless and autonomous communication. This decentralized structure ensures network robustness, fault tolerance, and adaptability, making AMNs well-suited for dynamic and unpredictable environments. A key advantage of AMNs is their ability to enhance connectivity in remote, disaster-stricken, or infrastructure-deficient areas. By forming self-sustaining relay networks, AMNs support mission-critical applications such as emergency response, environmental monitoring, and military surveillance. Specifically, AMNs wirelessly interconnect UAVs in a cooperative manner, maintaining communication links even in challenging conditions. This capability enables wide-area aerial coverage, making it suitable for large-scale deployment scenarios [30,31]. Figure 5 illustrates the architecture of AMN.

#### 2.1.4. UAV Swarm Networks

**Figure 6:**
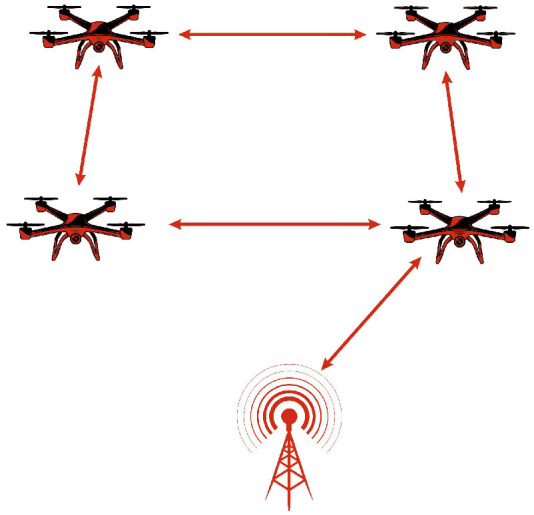
UAV swarm network architecture.

A UAV swarm is a coordinated group of UAVs that operate collectively to complete tasks with greater efficiency, minimal human intervention, and enhanced adaptability. Unlike FANETs, which focus on maintaining communication between UAVs for networked data transmission, UAV swarms emphasize cooperative behavior, distributed decision-making, and synchronized task execution. Inspired by natural swarm intelligence seen in flocks of birds or colonies of bees, these networks rely on decentralized control mechanisms, where individual UAVs react to local environmental inputs while maintaining overall swarm cohesion. While traditional swarm models depend on a central ground control station (GCS) for coordination, advanced swarm architectures leverage inter-UAV communication, allowing for self-organizing, resilient, and scalable operations. Depending on the level of autonomy, control can range from human-supervised coordination to fully autonomous operation, where onboard algorithms handle navigation, obstacle avoidance, and task distribution [32,33]. Figure 6 illustrates the architecture of UAV swarm network.

### 2.2. IBN

Infrastructure-based networks provide reliable connectivity, efficient resource management, and enhanced scalability, making them essential for a wide range of applications. These networks form the backbone of modern communication systems, including cellular networks, Wi-Fi infrastructure, and enterprise networking. They play a crucial role in smart cities, healthcare systems, and industrial automation by enabling efficient data transmission and seamless connectivity. Unlike infrastructure-less networks, which rely on direct device-to-device connections, infrastructure-based networks depend on access points (APs) to relay and manage network traffic, ensuring stable and high-speed communication across multiple users and regions [34,35]. In this article, we divided IBN into four subgroups: UAV-assisted cellular networks, UAV-based relay networks, UAV–Satellite Integrated Networks, UAV-SDN-Enabled Networks.

#### 2.2.1. UAV-Assisted Cellular Networks

**Figure 7:**
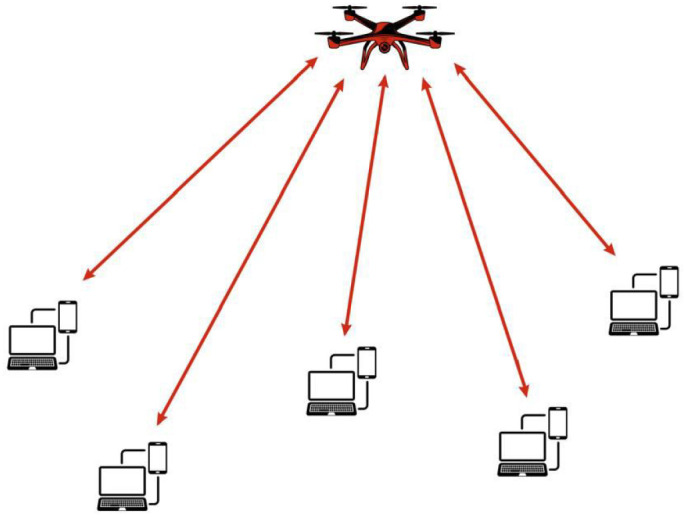
UAV-assisted cellular network architecture.

UAV-assisted cellular networks enhance coverage, capacity, and reliability by deploying UAVs as flying base stations or MEC nodes. Unlike traditional infrastructure reliant on ground-based towers, these networks provide adaptive connectivity in areas with limited or disrupted infrastructure. Their mobility and line-of-sight (LoS) communication improve spectral efficiency, reduce latency and support applications such as IoT, autonomous vehicles, and real-time surveillance. A key advantage is their ability to extend coverage in remote and disaster-stricken areas, ensuring seamless connectivity where terrestrial base stations are unavailable. UAVs dynamically adjust their positions based on traffic demand, optimizing performance and reducing congestion in urban environments. Integrating MIMO technology further enhances efficiency through spatial multiplexing, beamforming, and interference reduction. Additionally, UAV-assisted cellular networks support ultra-reliable low-latency communication (URLLC) and enhanced mobile broadband (eMBB), making them essential for 5G and beyond. For instance, during natural disasters, these networks restore emergency communications, aiding first responders with real-time data for faster disaster response [36,37]. Figure 7 illustrates the architecture of a UAV-assisted cellular network.

#### 2.2.2. UAV-Based Relay Networks

**Figure 8:**
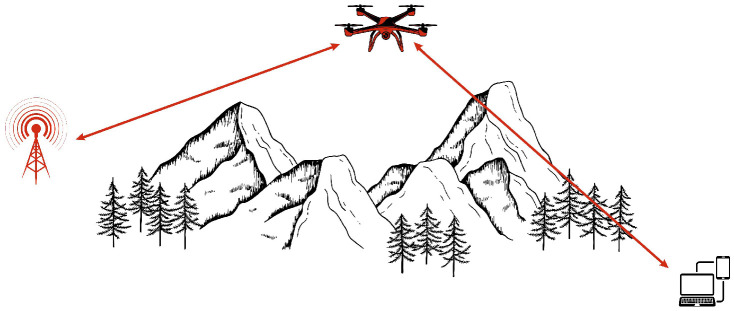
UAV-based relay network architecture.

Relay networks based on UAVs extend wireless coverage and improve connectivity in areas where direct communication links are unreliable or obstructed. Unlike static relays, UAVs dynamically adjust their positions to optimize coverage, enhance signal strength, and reduce interference, enabling long-range, high-speed data transmission. A key advantage is their ability to leverage LoS communication, minimizing the degradation of the signal from obstacles and terrain. This makes them valuable for disaster recovery, remote sensing, rescue operations, and rural connectivity. By forming multi-hop relay links, UAVs bridge communication gaps over long distances, ensuring uninterrupted connectivity. To enhance performance, UAV relay networks incorporate advanced techniques such as MIMO for improved data rates, millimeter wave (mmWave) communication for high capacity links, and simultaneous wireless information and power transfer (SWIPT) for energy efficiency. Increasingly integrated in next-generation networks, UAV relays work alongside high-altitude platform stations (HAPS) and low Earth orbit (LEO) satellites, leveraging AI-driven control for real-time network optimization [38,39]. Figure 8 illustrates the architecture of a UAV-based relay network.

#### 2.2.3. UAV–Satellite Integrated Networks

**Figure 9:**
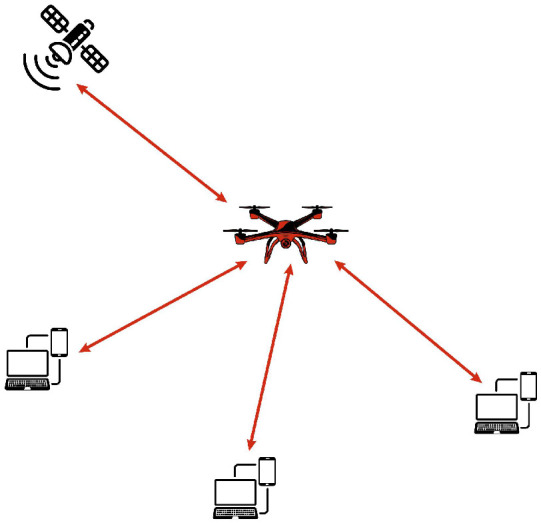
UAV–satellite integrated network architecture.

Communication between satellites and UAVs is essential for the realization of integrated space-air-ground networks. This link largely depends on a stable LoS connection and is particularly vulnerable to rain attenuation, especially when operating in higher frequency bands such as the Ka-band. Depending on their onboard equipment and mission requirements, UAVs can interact with satellites in different orbital regimes during flight. Geosynchronous satellites (GEO) are typically employed for satellite-to-UAV communication due to their fixed position relative to the Earth’s surface, which simplifies link maintenance. For the UAV-to-satellite uplink, successful communication relies on accurate alignment of the UAV’s transmission beam with the satellite. However, the continuous motion and changing orientation of UAVs can affect beam alignment, potentially degrading link quality. In typical UAV-assisted satellite communication scenarios, the UAV adjusts its beam direction dynamically to maintain a stable connection with the satellite as it navigates [40,41]. Figure 9 illustrates the architecture of a UAV–satellite integrated network.

#### 2.2.4. UAV-SDN-Enabled Networks

**Figure 10:**
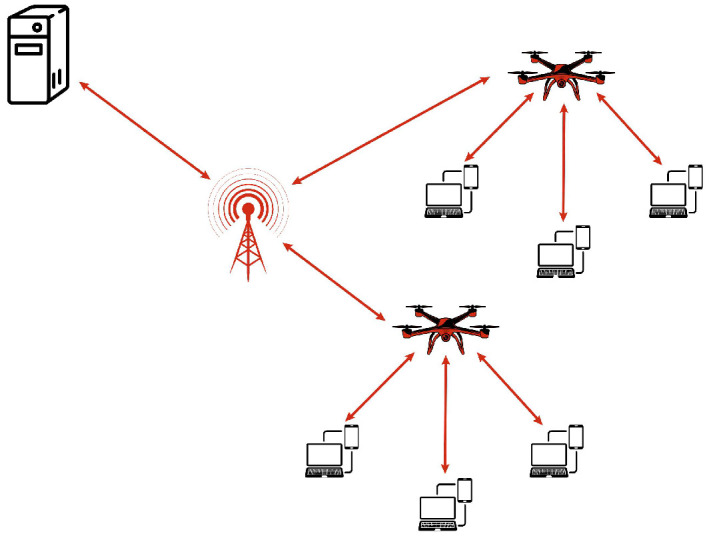
UAV-SDN-Enabled Network architecture.

UAV-SDN-Enabled Networks offer enhanced flexibility, agility, and resilience by integrating Software-Defined Networking (SDN) into aerial communication infrastructures. SDN introduces network programmability by decoupling the control and data planes through a logically centralized controller. This separation of concerns enables dynamic network reconfiguration, efficient resource allocation, and improved management of network infrastructure. As illustrated in Figure 10, UAVs operate as drone cells, providing wireless connectivity to ground users in a decentralized manner, especially in environments where terrestrial infrastructure is absent or insufficient. In this architecture, a central base station connected to a centralized controller or core network server manages the overall network functionality, enabling real-time decision-making and coordination. UAVs act as data plane nodes, responsible for collecting user data and environmental context, while the base station and server function as control plane entities, managing routing, bandwidth allocation, and connectivity decisions. This division supports the seamless integration and removal of UAVs within the network and enables adaptive service delivery in response to changing conditions. By leveraging SDN, such an architecture significantly enhances the adaptability, manageability, and performance of UAV communication networks, making them highly effective for dynamic, remote, or mission-critical scenarios [42,43].

## 3. UAV Node Placement Problem in UAVCN

### 3.1. System Model

In UAVCNs, UAVs operate at altitudes ranging from 100 m to 3 km and are equipped with wireless technologies such as LTE, WiFi, or LPWAN. Depending on the network type and deployment scenario, UAVs can serve as flying access points or localization anchors for randomly distributed ground users (GUs) [44].

**Figure 11:**
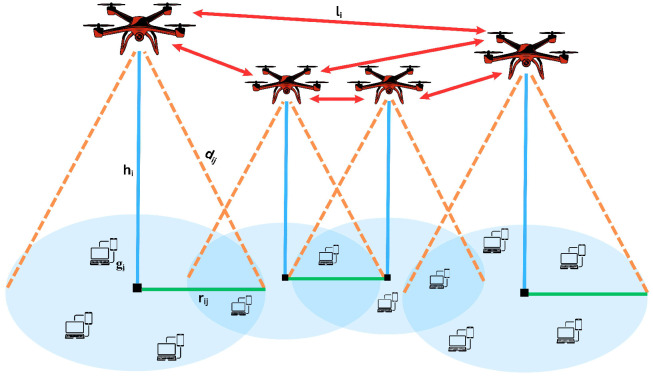
Communication between nodes via A2G and A2A links.

Usually, the system is represented by an undirected graph G=(V,E), where *V* is the set of all nodes and *E* is the set of communication links. The node set *V* consists of UAV nodes (UN) and ground users (GU). UAVs are deployed in 3D space and denoted as $UN=\{u_{1},u_{2},\ldots ,u_{i}\}$, where each UAV $u_{i}$ has coordinates $\left(x_{i},y_{i},h_{i}\right)$, with $h_{i}\in \left[h_{\min},\;h_{\max}\right]$ representing the UAV’s altitude. GUs are distributed on the 2D ground plane and denoted as $GU=\{g_{1},g_{2},\ldots ,g_{j}\}$, with each user $g_{j}$ located at $\left(x_{j},y_{j},0\right)$. The edge set *E* represents communication links, which include Air-to-Air (A2A) links between UAVs and Air-to-Ground (A2G) links between UAVs and ground users, established based on signal strength and coverage criteria. UAVs are often arranged in equilateral triangles in the air with adjustable side lengths $l_{i}$ and altitudes $h_{i}$ to ensure overlapping coverage areas, as illustrated in Figure 11 [45].

To model communication between UAVs and GUs, the Friis free-space path loss model is commonly used [46]. The received signal power $S_{ij}$ (in dBm) between a UAV $u_{i}$ and a ground user $g_{j}$ can be calculated as follows [46]:

(1) $$S_{ij}=P_{t}+G_{t}+G_{r}-20\log_{10}\left(\frac{4\pi f_{c}}{c}\right)-20\log_{10}\left(d_{ij}\right),$$

where $P_{t}$ is the transmit power (dBm), $G_{t}$ and $G_{r}$ are the antenna gains (dBi), $f_{c}$ is the carrier frequency (Hz), and *c* is the speed of light $\left(3\times 10^{8}\;m/s\right)$. The absolute LOS distance $d_{ij}$ is calculated as follows [46]:

(2) $$d_{ij}=\sqrt{h_{i}^{2}+r_{ij}^{2}},$$

where $r_{ij}$ is the Euclidean distance on the ground between $g_{j}$ and the projection of UAV $u_{i}$.

While the Friis model provides a simple and widely used approximation, it does not capture probabilistic line-of-sight (LoS) or non-line-of-sight (NLoS) propagation effects that may occur in urban, suburban, or obstructed environments. To account for more realistic A2G conditions, the channel can be modeled using a probabilistic combination of LoS and NLoS links. The average path loss between UAV $u_{i}$ and ground user $g_{j}$ can be expressed as follows [47]:

(3) $$PL_{ij}=P_{\mathrm{LoS},ij}\cdot PL_{\mathrm{LoS}}\left(d_{ij}\right)+\left(1-P_{\mathrm{LoS},ij}\right)\cdot PL_{\mathrm{NLoS}}\left(d_{ij}\right),$$

where $d_{ij}$ is defined in (2).

The probability of a LoS connection depends on the elevation angle between UAV $u_{i}$ and ground user $g_{j}$ and is given by:

(4) $$P_{\mathrm{LoS},ij}=\frac{1}{1+a\exp\left(-b(\theta_{ij}-a)\right)},$$

where *a* and *b* are environment-dependent parameters. The elevation angle $\theta_{ij}$ is defined as follows:

(5) $$\theta_{ij}=\arctan\left(\frac{h_{i}}{r_{ij}}\right),$$

with $h_{i}$ denoting the altitude of UAV $u_{i}$.

The path loss under LoS and NLoS conditions is modeled as follows [47]:

(6) $$PL_{\mathrm{LoS}/\mathrm{NLoS}}\left(d_{ij}\right)=20\log_{10}\left(d_{ij}\right)+20\log_{10}\left(f_{c}\right)+C_{\mathrm{LoS}/\mathrm{NLoS}},$$

where $C_{\mathrm{LoS}/\mathrm{NLoS}}$ represents the excessive path loss due to shadowing, diffraction, and blockage effects caused by the environment.

This system-level abstraction captures the impact of UAV altitude, link distance, and environmental conditions on signal attenuation and is sufficient for analyzing coverage, connectivity, and UAV node placement in UAV communication networks.

### 3.2. Placement Objectives

The node placement problem in UAVCNs is a critical multi-objective optimization task driven by the need to enhance overall network performance while considering dynamic topologies, energy constraints, QoS requirements, and UAV altitude. Optimal placement strategies balance the horizontal positioning and altitude of UAVs to maximize key performance metrics such as user coverage, node connectivity, and throughput while minimizing end-to-end delay, energy consumption, and the packet delivery ratio (PDR) to support sustained and scalable operations. To quantitatively evaluate these objectives, standard performance metrics are typically expressed using well-established mathematical formulations, as presented below [45,46,47,48,49,50,51].

#### 3.2.1. Coverage

It refers to the spatial extent within which UAVs can reliably transmit communication signals to ground users and can be defined as follows [45]:

(7) $$\psi \left(G\right)=\sum_{i=1}^{n}\left(\max_{j\in \{1,\ldots ,m\}}\sigma_{ij}\right),$$

where the coverage variable $\sigma_{ij}$ is represented as follows:

(8) $$\sigma_{ij}=\left\{\begin{array}{cc} 1, & \mathrm{if}\;S_{ij}\geq \gamma ,\\ 0, & \mathrm{otherwise}. \end{array}\right.$$

here, $S_{ij}$ denotes the received signal power as defined in (1), and γ is the minimum received power threshold required for reliable communication.

#### 3.2.2. Connectivity

It refers to the ability of nodes within UAVCN to establish and maintain reliable communication links with one another, ensuring continuous data exchange across the network. It is calculated as follows [45]:

(9) $$\Phi \left(G\right)=\max_{i\in \{1,\ldots ,k\}}\left|G_{i}\right|,$$

where $|G_{i}|$, i∈{1,…,k}, is the number of nodes in the *i*th connected subgraph, and $G=G_{1}\cup G_{2}\cup \ldots \cup G_{k}$.

#### 3.2.3. Throughput

It refers to the volume of data successfully transmitted across a communication channel within a specified time interval. The throughput *T* in bits per second is calculated as follows [49]:

(10) $$T=\frac{\sum_{i=1}^{n}R_{ij}\times \mathrm{Packet}\;\mathrm{Size}}{T_{\mathrm{sim}}},$$

where $R_{i}j$ is the number of packets successfully received by node *j* from node *i*, PacketSize is in bits, and $T_{\mathrm{sim}}$ is the simulation time in seconds.

#### 3.2.4. End-to-End Delay

It refers to the time required for a data packet to travel from the source node to the destination node. End-to-end delay can be defined as follows [48]:

(11) $$\text{End-to-End}\;\mathrm{Delay}=\frac{1}{n}\sum_{i=1}^{n}\left(p_{i}\left(T_{r}\right)-p_{i}\left(T_{s}\right)\right),$$

where $p_{i}$ is the *i*th packet, $p_{i}\left(T_{s}\right)$ is the time at which the packet was transmitted, $p_{i}\left(T_{r}\right)$ is the time at which the packet was received, *n* is the number of successfully delivered packets.

#### 3.2.5. Energy Consumption

It refers to the amount of energy used by UAVs during operation, including both communication and flight energy costs. Efficient energy modeling is essential for optimizing deployment and extending UAV network lifetime. A realistic model of the energy consumed by UAV $u_{j}$ during flight is given by [50]:

(12) $$E_{j}=\left(\alpha +\beta z_{j}\right)\cdot T+P_{\mathrm{Max}}\cdot \left(\frac{z_{j}}{S_{j}}\right),$$

where α is the minimum power required to maintain flight, β is a motor speed multiplier, *T* is the flight time, $P_{Max}$ is the maximum motor power, and $z_{j}$ and $S_{j}$ denote the altitude and speed of UAV $u_{j}$, respectively.

#### 3.2.6. Packet Delivery Ratio (PDR)

It represents the reliability of data transmission across the network. The PDR can be mathematically defined as follows [51]:

(13) $$\mathrm{PDR}=\left(\frac{\sum RP_{d}}{\sum SP_{s}}\right)\times 100,$$

where $\sum RP_{d}$ is the number of packets received at destination nodes, and $\sum SP_{s}$ is the number of packets sent by source nodes.

## 4. Metaheuristic Algorithms in UAV Optimization

This section presents an overview of MHAs as a promising approach to solving the UAV node placement problem [52,53,54,55,56,57,58,59,60,61]. The emphasis is placed on their key advantages over conventional optimization methods, as well as the fundamental principles that govern their operation. Over the past decade, MHAs have gained widespread popularity due to their effectiveness in tackling NP-hard problems and their ability to efficiently explore complex, high-dimensional search spaces. They are recognized as computationally efficient tools capable of delivering high-quality solutions while maintaining robustness and ensuring convergence [62,63,64,65,66,67,68,69,70,71].

Compared to traditional optimization techniques, which often struggle with the computational demands of NP-hard problems, MHAs offer several distinct benefits, including ease of implementation, global search capability, effective constraint handling, and suitability for parallel processing. As a class of high-level optimization strategies, meta-heuristics are specifically designed to find near-optimal solutions in cases where exact methods are impractical due to the vast size or complexity of the solution space [72,73,74,75,76,77].

Many of these algorithms draw inspiration from natural phenomena, such as evolutionary biology [78,79,80,81,82,83], swarm behavior [84,85,86,87], or neighborhood-based search patterns. Originating from basic heuristic methods rooted in trial-and-error, meta-heuristics have evolved to incorporate both randomization and local search techniques, thereby enhancing their adaptability and performance across a wide range of optimization tasks [88,89,90].

Two key components characterize meta-heuristics: intensification, which focuses the search around promising regions, and diversification, which introduces randomness to explore the broader solution space and avoid premature convergence to local optima. Although they do not guarantee discovery of the global optimum, meta-heuristics are highly effective at delivering high-quality, feasible solutions within practical computational limits [91,92].

**Figure 12:**
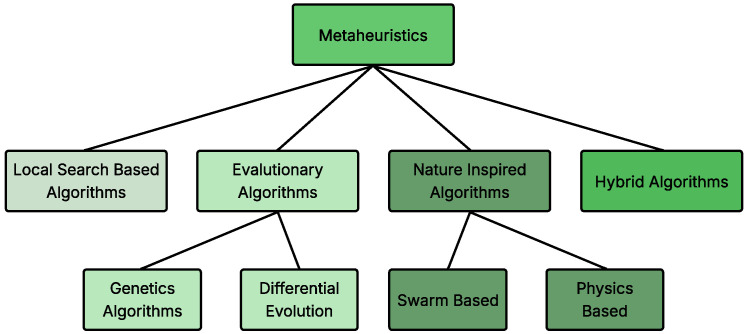
Classification of meta-heuristic algorithms.

Due to their balance between solution quality and computational efficiency, meta-heuristics have been widely adopted across various fields [93,94,95,96]. In this article, MHAs are categorized into four groups: Local Search-Based Algorithms, Evolutionary Algorithms, Nature-Inspired Algorithms, and Hybrid Algorithms, as illustrated in Figure 12.

### 4.1. Evolutionary Algorithms

Evolutionary Algorithms (EAs) are population-based optimization techniques that leverage Darwin’s theory of natural selection to guide the search process. These meta-heuristic approaches are designed to solve complex optimization problems that are not easily addressed using traditional linear programming techniques. EAs operate on the principle of biological evolution, where a population of potential solutions is generated and iteratively refined over successive generations. The effectiveness of each solution is evaluated using a fitness function, which quantifies its quality. Through repeated variation and selection, the population evolves toward improved solutions [97]. Evolutionary algorithms are primarily categorized into two main branches: Genetic Algorithm (GA) and Differential Evolution (DE). Among these, GA is the most widely utilized and is recognized as one of the most effective methods for solving a wide range of optimization problems.

**Figure 13:**
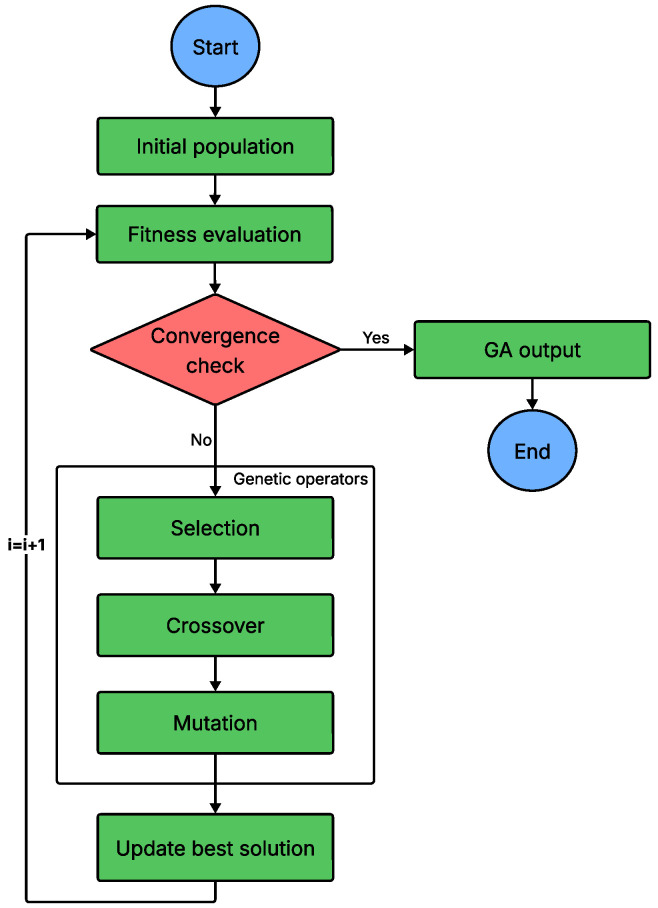
Flowchart of GA.

GA is a well-established meta-heuristic inspired by natural selection and the principle of “survival of the fittest.” Introduced by J.H. Holland [98], GA evolves a population of candidate solutions using genetic operators such as selection, crossover, and mutation. Solutions are typically encoded as binary strings, with each chromosome representing a point in the search space. A fitness function evaluates the quality of each chromosome, guiding the evolutionary process. Starting from a randomly initialized population, successive generations are produced by preferentially selecting fitter individuals, recombining their genetic material through crossover, and introducing mutations to maintain diversity and avoid premature convergence. GA often employs adaptive mechanisms to adjust crossover and mutation rates dynamically, balancing exploration and exploitation. Its ability to perform global search and evaluate multiple solutions in parallel makes GA a versatile tool for complex optimization problems across various domains [99]. The algorithm that summarizes this working principle is presented in Figure 13.

**Figure 14:**
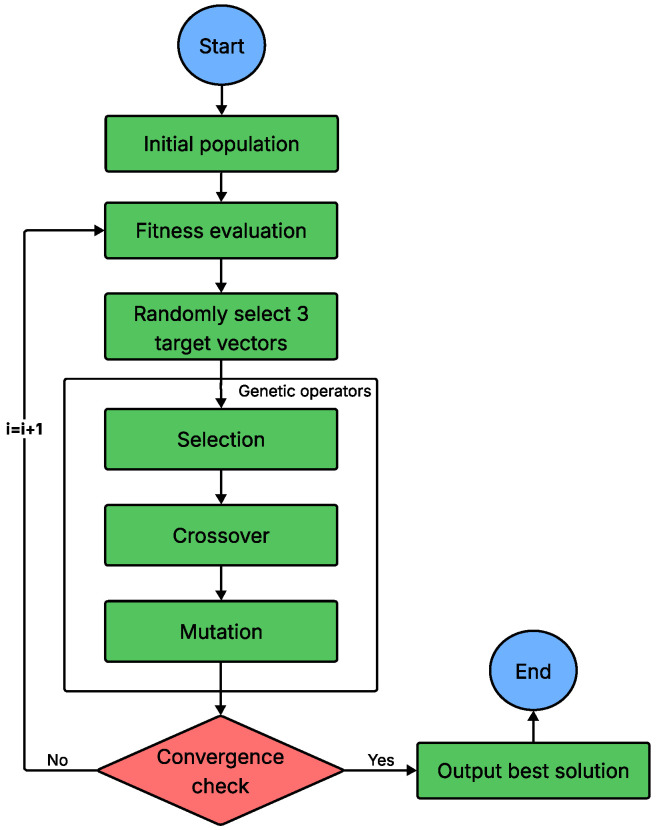
Flowchart of DE.

DE, introduced by Storn and Price [100], is another population-based evolutionary algorithm that shares an iterative structure with GA but relies on different variation operators. In each generation, DE creates a mutant vector by adding the scaled difference between two randomly chosen population vectors to a third vector; it then performs crossover to blend this mutant with the current candidate, forming a trial vector. Selection chooses the solution with the better objective value between the parent and the trial to carry forward to the next generation. This simple yet powerful cycle repeats until a predefined stopping criterion is satisfied. Owing to its simplicity, robustness and adaptability, DE is now a standard tool for solving complex global-optimization problems in science and engineering [101]. The step-by-step algorithm of DE is summarized in Figure 14.

### 4.2. Nature-Inspired Metaheuristic Algorithms

Nature-Inspired Metaheuristic Algorithms (NIMAs) are optimization techniques inspired by natural phenomena, designed to solve complex, non-linear problems through iterative exploration and exploitation of the solution space. They often use populations of simple agents whose collective behavior leads to effective search strategies. Yang [102] played a key role in formalizing many of these algorithms, outlining their structures and applications. NIMAs are typically classified into swarm-based and physics-based algorithms, each mimicking behaviors like animal swarming or physical laws to guide the search toward optimal solutions [103].

**Figure 15:**
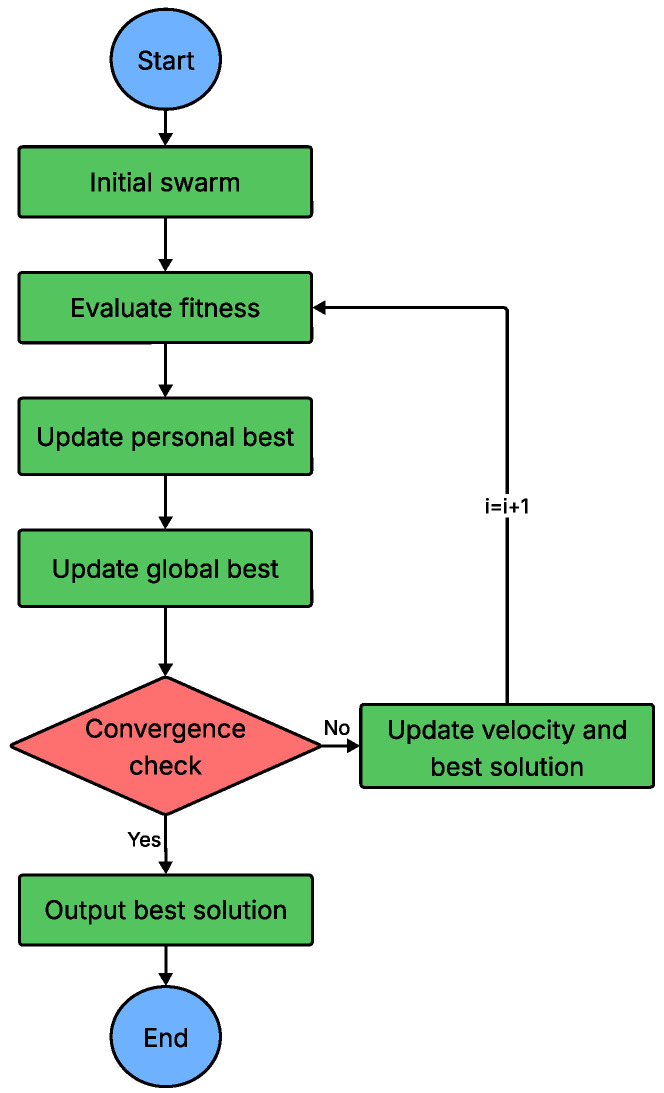
Flowchart of swarm-based NIMA.

Swarm-based NIMAs are optimization techniques modelled on the collective behavior of social organisms such as insects, birds and fish. First proposed by Beni and Wang [104] for cellular robotic systems, swarm intelligence (SI) has matured into a powerful paradigm for tackling complex search problems. A hallmark of SI is its decentralized control: simple agents interact only locally and obey elementary behavioral rules, yet intelligent global behavior emerges through self-organization and division of labor. Owing to this property, swarm-based NIMAs offer simplicity, flexibility and strong global-search capability, making them well suited to dynamic or large-scale optimization tasks. Their applications include path planning, scheduling, clustering, load balancing, wireless communication, IoT systems and robotics [105]. The generic workflow of a swarm-based NIMA is outlined in Figure 15.

**Figure 16:**
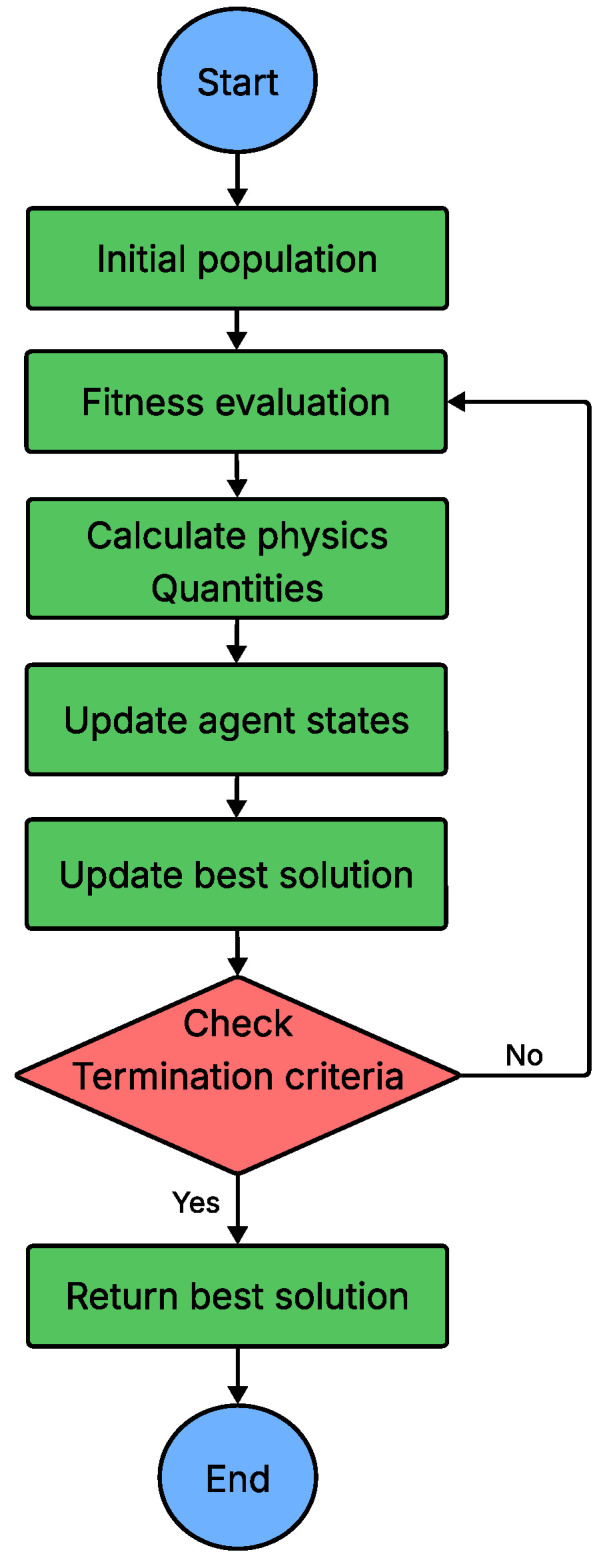
Flowchart of physics-based NIMA.

Physics-based NIMAs, in contrast, are inspired by physical laws and processes such as gravitational attraction, thermal motion, energy dissipation, and equilibrium dynamics, which they use to iteratively refine candidate solutions. One of the earliest and best-known examples is SA, which mimics the controlled cooling of a material to probabilistically escape local optima and approach a global optimum. More generally, these algorithms emulate systems governed by physical forces, thereby maintaining an effective balance between exploration and exploitation. By abstracting physical behaviour into computational form, physics-based NIMAs provide robust, scalable approaches for tackling complex and nonlinear problems in engineering design, control systems and machine learning [106]. The overall workflow of a typical physics-based NIMA is depicted in Figure 16.

### 4.3. Local-Search-Based Algorithms

**Figure 17:**
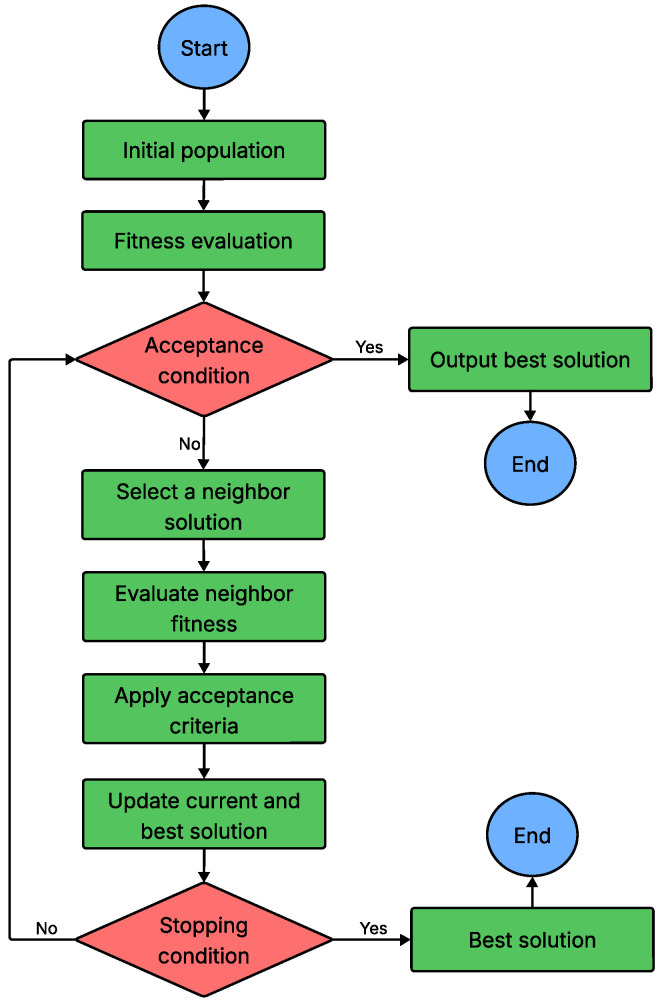
Flowchart of local-search-based MHA.

Local-search-based algorithms are commonly employed to solve combinatorial optimization problems, where the objective is to identify the best solution within a finite set of possibilities. These algorithms begin with an initial solution and iteratively improve it by applying small modifications called neighborhood operations. The neighborhood consists of all solutions that can be reached from the current solution through such changes. When none of the neighboring solutions offer an improvement, the current solution is deemed a local optimum. However, this local optimum may not represent the global best solution. To overcome the challenge of becoming trapped in local optima, various techniques have been developed. These include accepting worse solutions temporarily through random changes, avoiding recently visited solutions, and exploring different neighborhood structures. By employing these strategies, local search algorithms enhance their exploration capabilities and improve the likelihood of finding the global optimum [107]. The general workflow of a local-search-based meta-heuristic algorithm is summarized in Figure 17.

### 4.4. Hybrid Meta-Heuristic Algorithms

**Figure 18:**
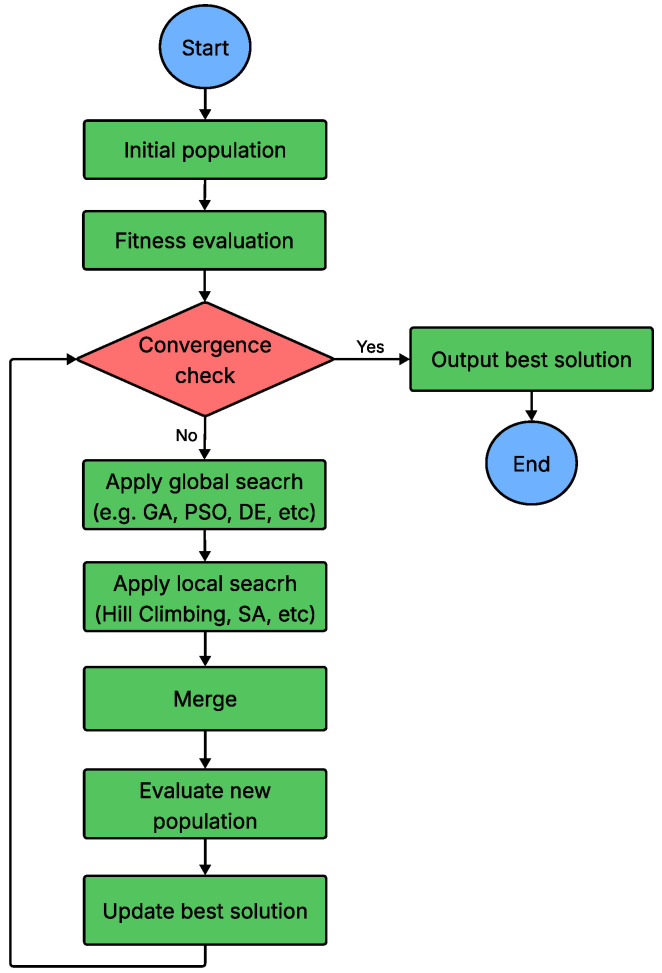
Flowchart of hybrid MHA.

Hybrid meta-heuristic algorithms represent a promising research direction that combines the strengths of multiple nature-inspired meta-heuristics to overcome the limitations of individual methods and enhance overall optimization performance. By integrating complementary strategies such as the global exploration capabilities of swarm-based algorithms and the local exploitation strengths of physics-based methods, hybrid models achieve a more balanced and effective search process. This balance improves convergence speed, solution accuracy, and robustness. For example, swarm intelligence techniques like PSO excel at rapid global exploration, while physics-based methods such as SA provide powerful mechanisms to escape local optima through controlled randomization. A hybrid approach leverages the exploratory power of one algorithm while refining solutions using the intensification mechanisms of another. These combinations can be implemented through parallel, sequential, or cooperative hybridization strategies. In UAV communication networks, hybrid meta-heuristics have demonstrated significant potential for solving complex problems, including energy-aware node placement, delay-sensitive task offloading, and adaptive routing. Overall, the hybridization paradigm not only enhances algorithmic flexibility and adaptability but also leads to the development of more generalizable and efficient optimization solvers applicable across a wide range of domains [108]. The step-by-step procedure of a typical hybrid meta-heuristic algorithm is outlined in Figure 18.

## 5. Metaheuristic Algorithms for UAV Placement: Open Problems and Future Challenges

This section presents an analysis of meta-heuristic algorithms applied to the UAV node placement problem. The discussion is organized by algorithmic families, such as nature-inspired meta-heuristics, evolutionary algorithms, hybrid approaches, and local-search-based methods, highlighting the key strengths and current limitations of each category. Open research problems and potential directions for future enhancement are also identified in order to support the development of more robust and adaptive solutions for UAV network deployment.

### 5.1. GA Based Algorithms

GAs have been widely adopted in UAV communication networks due to their global search capability, adaptability to various constraints, and suitability for complex multi-objective optimization problems. Their population-based nature and stochastic operators allow for efficient exploration of large, nonconvex search spaces, making them effective for UAV placement in both centralized and distributed architectures.

In [52], a GA optimized UAV topology for throughput enhancement in FANETs, treating UAVs as routers forming a dynamic wireless mesh. The algorithm used a tailored encoding and adjacency-based fitness function while enforcing movement constraints via the Radius of Position Constraint (RPC) and Radius of Particle Size (RPS). The results showed that increasing RPC enhanced throughput by expanding search space, while lower RPS yielded finer control. While promising, the study assumes a fixed UAV altitude and ideal wireless conditions, which may limit its generalizability in more variable real-world scenarios.

In [79], a GA was used to jointly optimize UAV placement and user association in a high-altitude platform (HAP)-UAV hybrid system. Initial UAV positions were estimated using K-means clustering to enhance convergence speed. The GA-based solution improved throughput by 32.5% compared to the HAP-only baseline under a 100 m user distribution radius and maintained flexibility by enabling global user association. While effective in wide-area and sparse deployments, the method relied on perfect LoS assumptions and static users.

In [78], a Multi-Layout Multi-Subpopulation GA (MLMPGA) was proposed for UAV placement with multi-objective optimization of coverage, fault-tolerance, and redundancy. Simulations with 50–125 ground nodes and 10–18 UAVs showed that MLMPGA outperformed standard GA, PSO, and Hill Climbing in more than 80% of scenarios. It achieved the best coverage and fault-tolerance for 125 nodes with 14 UAVs, and superior redundancy for 75 nodes with 18 UAVs. Its adaptive layout-based evolution, using distinct mutation and crossover rates per subpopulation, improved the exploration–exploitation balance and avoided local optima. However, the increased computational cost may limit real-time use, favoring offline optimization.

In [96], an optimization framework for Flying Backhaul Networks (FBNs) employed Nondominated Sorting Genetic Algorithm II (NSGA-II) for UAV placement and an inner GA for routing. Simulations over a 500 × 500 m^2^ area with 24 ground nodes and traffic loads from 30–150 kbps demonstrated that NSGA-II produced Pareto-optimal placements. To support a 720 kbps load, only four UAVs were required, while two sufficed for 240 kbps. The strategy consistently maintained average PDRs above 0.8, even under higher loads. These results emphasize the framework’s ability to minimize infrastructure while ensuring reliable, interference-aware UAV deployments under dynamic traffic demands. However, the framework does not consider UAV mobility or time-varying topologies, limiting its applicability to dynamic FBN scenarios.

Generally, GA-based algorithms are highly effective in maximizing coverage and throughput, reducing average delay, enabling adaptive topology control, and supporting coordinated UAV deployments across various scenarios. Their flexibility and strong performance in solving joint optimization tasks make them well-suited for scalable UAV networks. In addition, GAs can accommodate heterogeneous UAV types and heterogeneous traffic demands, which is essential for practical deployments involving both aerial and terrestrial users.

To further enhance deployment realism, future research should incorporate energy constraints, interference modeling, and mobility-aware optimization. Integration with machine learning models to predict traffic patterns and environmental dynamics can also improve responsiveness in real-time UAV placement. Moreover, combining GAs with other techniques like reinforcement learning or game theory could help in addressing the dynamic nature of aerial networks while maintaining scalability.

### 5.2. DE-Based Algorithms

DE is a robust evolutionary optimization technique known for its simplicity, fast convergence, and effectiveness in solving complex, multi-dimensional problems. In the context of UAV node placement, DE has been successfully applied to optimize deployment strategies in dynamic and large-scale environments. It performs well in continuous search spaces, requires few control parameters, and avoids premature convergence better than some traditional evolutionary methods.

In [54], the authors proposed DEVIPS, a DE variant with Variable Population Size, designed for energy-efficient UAV deployment in IoT data collection systems. By converting the variable-length optimization problem into a fixed-length representation, DEVIPS enabled the use of traditional DE operators while maintaining adaptability across different scenarios. It achieved up to 17.44% lower energy consumption compared to other evolutionary algorithms such as VLGA, fGA, and JGGA. However, the use of fixed-length encoding may reduce flexibility in environments with frequently changing distributions of ground nodes.

In [83], DE was applied in a joint UAV placement and task scheduling framework for a multi-UAV MEC architecture. While DE optimized UAV positions, a Deep Reinforcement Learning (DRL) module managed task scheduling. The DE algorithm achieved the best deployment fitness score (−5.0) within 50 iterations, outperforming GA and PSO. However, its reliance on near-optimal task assignment strategies can introduce scalability challenges in highly dynamic or large-scale IoT scenarios.

In [22], the DE was applied to optimize the contention window (CW) size in FANETs, yielding notable numerical gains. Under CSMA/CA, DEA achieved a throughput of 7.481 Mbps with 10 UAVs, outperforming the traditional MAC’s 7.0797 Mbps. At 30 UAVs, DEA sustained a throughput of 7.4089 Mbps, and when the UAV count exceeded 35, it recorded the lowest packet drop rate (PDR) among the tested algorithms. DEA also demonstrated faster computation than CUCO and HBA. These results confirm DEA’s effectiveness for real-time MAC parameter tuning in dense UAV networks.

Generally, DE-based approaches show strong performance in optimizing UAV placement for energy efficiency, balanced task distribution, and adaptability to complex deployment conditions. Their differential mutation and crossover mechanisms make them well-suited for exploring continuous and high-dimensional search spaces. DE variants have also demonstrated robustness in handling multi-objective formulations, such as jointly minimizing energy consumption and maximizing coverage or link reliability. Moreover, DE-based algorithms are particularly effective in achieving good coverage and connectivity, and have shown respectable throughput performance, especially in scenarios requiring adaptive topology formation under resource constraints.

To improve their practicality in real-time and dynamic networks, future research should explore hybrid strategies that integrate DE with online learning, real-time adaptation, and consideration of UAV constraints such as mobility and coordination overhead. In addition, incorporating environment-sensitive mechanisms, such as dynamic obstacle avoidance, channel fading models, and traffic pattern prediction, can significantly enhance the realism and robustness of DE-based UAV deployment strategies.

### 5.3. Swarm-Based Algorithms

Swarm-based algorithms such as PSO and GWO are frequently applied in UAV communication networks due to their ability to efficiently explore large solution spaces and adapt to dynamic topologies. Their decentralized structure and fast convergence make them particularly suitable for time-sensitive and mobile scenarios where centralized control is impractical.

In [70], a PSO-based topology formation strategy was introduced for FANETs, prioritizing graph-theoretic metrics like node connectivity and bridge count rather than traditional distance-based criteria. This approach significantly enhanced fault tolerance by increasing the average node degree from 3.5 to 5, eliminating network bridges, and maintaining full connectivity in all simulation runs while reducing packet loss by 10–80%. However, increased connectivity led to higher interference levels, reflected by rises in maximum clique size and chromatic number.

In [65], a PSO-based algorithm was proposed for dynamic 3D placement of LTE drone-mounted base stations serving machine-type communication devices (MTCDs). The method improved system throughput from approximately 15,000 Kbit/s to over 28,000 Kbit/s and reduced the deadline-missing ratio from 66% to below 36% while achieving a mean SNR around −2.3 dB and lowering computational complexity, making it suitable for real-time deployment. Despite these advantages, the algorithm exhibited performance degradation under highly dense or dynamic conditions due to limited coordination capabilities.

In [60], a GWO-based method was proposed for 3D placement of drone-mounted base stations in 5G networks to maximize downlink coverage. Using stochastic geometry to model SINR, the approach identified optimal drone positions under a coverage constraint. Simulations with 200 users in a 2 × 2 km^2^ area showed that 5 drones achieved 76% coverage, while 10 ensured full coverage. The method reduced infrastructure needs while maintaining high coverage probability, reaching up to 82.5%. However, effects such as signal blockage and drone overlap were not considered and are suggested for future research.

In [88], a PSO-based algorithm was applied for UAV base station placement in open areas using the Hata–Okumura model for path loss estimation. The method determined optimal drone positions and quantities based on antenna ranges. With eight UAVs, coverage improved from 26% (200 m range) to 69% (400 m) and 87% (500 m), efficiently covering an 8 km^2^ area. Higher antenna ranges eliminated the need for additional drones. The study also analyzed path loss over 800–1500 MHz, showing that higher frequencies increased loss over long distances. However, the method may be less effective in environments with obstacles and rapidly changing channel conditions.

Generally, swarm-based algorithms demonstrate strong scalability and adaptability across various deployment sizes, offering fast and practical solutions for real-time UAV placement tasks. They are particularly effective in enhancing network connectivity, coverage, and throughput, making them highly suitable for mobile and emergency UAV scenarios, such as disaster recovery and temporary hotspot coverage. Their decentralized nature and self-organizing behavior enable efficient handling of dynamic topologies and intermittent links. However, swarm algorithms may be less ideal for latency-sensitive applications due to potential convergence delays and limited fine-tuning capabilities in dense or highly constrained environments.

To further improve their applicability, future research could focus on incorporating interference-aware models, real-time adaptive control, and hybrid techniques that combine swarm intelligence with local refinements, meta-learning, or deep reinforcement learning. Integrating mobility prediction and task-aware placement strategies can improve responsiveness and resource allocation. Additionally, enhancing energy efficiency, inter-UAV coordination, and robustness to adversarial conditions (e.g., jamming, obstacles, and packet loss) will be essential for deploying swarm-based solutions in next-generation UAV networks operating under high mobility and environmental complexity.

### 5.4. Physics-Based Algorithms

Physics-based algorithms, such as Simulated Annealing (SA) and Deterministic Annealing (DA), are particularly effective for solving highly nonlinear and constrained optimization problems such as UAV placement. They are well suited for scenarios where flexible adaptation to dynamic conditions is required, such as in dense urban networks, wide-area ad hoc deployments, or temporary emergency setups. These algorithms offer a balanced trade-off between solution quality and computational cost, making them a practical choice for complex environments where UAVs are required to operate reliably under unpredictable constraints.

In [61], an SA-based algorithm was applied for 3D placement of multiple UAV-BSs to maximize coverage, avoid collisions, and meet user data rate requirements. The AI-driven approach dynamically adjusted UAV positions, enabling up to 95% of users to meet QoS targets, compared to 75% with static placement, and boosted throughput from $1.2\times 10^{9}$ to over $2.2\times 10^{9}$ bit/s for 500 users. However, the proposed method does not consider UAV hovering time limitations or user mobility, which may affect performance in dynamic environments.

In [64], the UAV placement problem in wide-area ad hoc networks was formulated as a clustering task using a distortion-based cost function and solved using Deterministic Annealing. The goal was to minimize the number of UAVs while maintaining full network connectivity. Simulations with 15 ground nodes showed that full connectivity can be achieved with only 5 UAVs. When additional constraints were introduced, such as covering at least six nodes per cluster or ensuring redundancy with two to three UAVs per cluster, the required number increased to six or seven. However, the method may face limitations when applied to larger-scale networks with higher node density or complex terrain variability.

In [73], energy-efficient resource allocation was addressed in a UAV-assisted full-duplex NOMA system supporting URLLC under finite blocklength constraints. The joint optimization of 3D UAV placement and power allocation was solved using a hybrid method combining SA and convex approximation. The algorithm achieved fast convergence and showed up to 90 percent improvement over HD-NOMA and OMA baselines. It approached infinite blocklength performance while using short packets and maintaining low latency, making it suitable for mission-critical and delay-sensitive applications.

In [81], SA was applied to optimize the number and placement of UAV base stations for large-area 5G coverage. SA demonstrated reliable convergence in medium-scale areas, achieving full coverage with 6 UAVs for $80\;{\mathrm{km}}^{2}$, each covering around $10\;{\mathrm{km}}^{2}$. However, SA required longer execution times (up to 1.2 s) and showed slower convergence compared to Genetic Algorithm (GA). Its performance degraded slightly in very large areas (less than $44\;{\mathrm{km}}^{2}$) due to a higher chance of local optima. Despite this, SA maintained acceptable coverage efficiency and produced consistent layouts under constraints, indicating its suitability for moderate-scale, time-tolerant UAV deployment scenarios.

Generally, physics-based algorithms offer a powerful framework for UAV placement under complex constraints, particularly in optimizing energy efficiency and latency-aware design. Inspired by physical phenomena such as gravitational forces, electromagnetism, or thermal dynamics, these algorithms can produce high-quality, near-optimal solutions with moderate computational cost. Their continuous space search capability and sensitivity to constraint dynamics make them well suited for fine-grained placement tasks, such as ensuring balanced coverage in urban or heterogeneous environments. However, they are not ideal for highly time-critical applications without enhancements such as warm-starting, hybridization with local search, or incorporation of predictive models to accelerate convergence.

Future work should focus on integrating real-time system feedback, adaptive load balancing, and energy-aware UAV control strategies to support scalable and resilient UAV networks under practical deployment conditions. Additionally, embedding physics-based models with mobility prediction, environment-aware path planning (e.g., obstacle fields or no-fly zones), and multi-objective optimization (e.g., trade-offs between coverage, latency, and energy) can significantly boost their robustness. Leveraging parallel or distributed implementations can further reduce execution time, enabling applicability in real-time and large-scale scenarios.

### 5.5. Local-Search-Based Algorithms

Local-search-based algorithms are particularly effective in constrained environments where rapid convergence to high-quality solutions is necessary. They are well-suited for centralized control architectures and scenarios with limited UAV resources, enabling fine-grained adjustments and deterministic improvements. Local search techniques like tabu search, hill climbing, and adaptive neighborhood search can be easily combined with global optimization methods or learning-based strategies to overcome local optima and enhance robustness. Their simplicity, flexibility, and low computational overhead make them practical for time-sensitive or resource-constrained UAV communication applications.

In [66], an adaptive local-search-based Arithmetic Optimization (LSAO) algorithm was proposed for UAV placement in MANETs. Combining adaptive switching, chaotic local search, and opposition-based learning, LSAO effectively balances exploration and exploitation. It achieves up to 100% coverage and connectivity across multiple test cases, with 87.02% coverage and a fitness of 36.57 using 10 UAVs. While delivering strong performance with limited UAVs, the algorithm shows moderate efficiency in energy use and load distribution.

In [90], a centralized UAV positioning algorithm was proposed for SDN-based UAV networks to maximize system throughput using a tabu search based technique. Leveraging flow demand and path data from the SDN controller, the algorithm iteratively optimizes UAV locations, achieving 10–67% throughput gains with an average of 26%. It reaches 95% of the optimal throughput in 190 iterations and 99% by 870. However, its performance declines in dynamic settings due to sensitivity to user mobility, requiring re-execution when throughput drops by 30%.

Generally, local-search-based algorithms offer high precision and interpretability in UAV placement tasks, particularly under centralized control and moderate-scale scenarios. They are especially effective at improving local performance metrics such as coverage or throughput with low computational overhead and fast convergence. While they can contribute to improvements in connectivity and latency, their effectiveness is often limited in highly dynamic or large-scale networks due to their tendency to converge to local optima. Nonetheless, their simplicity and responsiveness make them practical for constrained UAV communication environments.

To further improve their applicability, future research could explore hybrid frameworks that integrate local search with reinforcement learning or meta-heuristic strategies, as well as enhancements for handling dynamic mobility patterns, energy constraints, and real-time responsiveness in large-scale UAV networks.

### 5.6. Hybrid Algorithms

Hybrid algorithms are effective in solving complex UAV placement problems by combining the strengths of multiple optimization techniques. This integration enables a balance between global exploration and local refinement, resulting in improved solution quality, faster convergence, and more effective handling of multi-objective trade-offs. These algorithms offer enhanced robustness across diverse scenarios, including high mobility and dynamic network topologies, while maintaining consistent performance. Moreover, their adaptability allows for real-time adjustments to fluctuating network conditions, varying user demands, and UAV-specific constraints, making them particularly suitable for mission-critical and time-sensitive applications.

In [20], HGA-SA and HWWO-HSA, two hybrid algorithms, were introduced for placement of 3D UAVs in post-disaster networks, targeting optimal coverage, connectivity and resilience. By incorporating Taguchi parameter tuning and graph-based strategies, HGA-SA outperformed GA and HS by up to 12% in small-scale scenarios, while HWWO-HSA achieved a 23.2% utility gain in larger deployments. However, this improvement came at the cost of an 18.4% increase in computational time, making them less ideal for time-critical applications.

In [15], the authors proposed IMRFO-TS, a hybrid of Improved Manta Ray Foraging Optimization and Tabu Search, tailored for smart city UAV deployments. The algorithm balances energy use and load distribution while maintaining high coverage and connectivity. Tested across 52 scenarios, IMRFO-TS achieved up to 99.1% coverage, full connectivity, and minimal energy consumption, consistently outperforming eight baseline methods. A minor limitation of the approach is its relatively high computational complexity, which could restrict its effectiveness in real-time applications.

In [46], the authors proposed BR-ILS, a hybrid Iterated Local Search algorithm designed to solve the UAV placement problem formulated as a single-allocation p-hub median optimization. The method aims to maximize link capacity and optimize UAV positioning for efficient ground user coverage. Tested on various instance sizes (up to 200 users and 10 UAVs), BR-ILS consistently achieved near-optimal results in significantly reduced computation times compared to the CPLEX solver, showing up to 12.24% improvement over naive heuristics. Despite its strengths in scalability and solution stability, the method may be less effective for very small instances where exact solvers like CPLEX, given sufficient time, can produce superior results.

In [93], a hybrid PSO with Simulated Annealing (HPSO) was applied for UAV-borne IRS placement in mmWave multicast systems. The method jointly optimizes UAV positions and beamforming to maximize the minimum rate for user clusters. Simulations at 28 GHz with three user clusters showed that HPSO achieved a 24.6% higher minimum rate than conventional PSO and 32.8% over random placement. With 128 IRS elements, the minimum rate reached 2.4 Gbps, compared to 1.5 Gbps with 64 elements. The approach required only 40 iterations to converge versus 100 for exhaustive search, significantly reducing computation. These results demonstrate the effectiveness of integrating SA into PSO for robust, fair, and efficient UAV placement in high-frequency environments. Despite this improvement, the method’s performance may degrade with a rapidly changing user distribution, requiring real-time adaptation.

Generally, hybrid algorithms demonstrate superior performance across diverse deployment scenarios, offering robust and scalable solutions for multi-objective UAV placement. By combining the strengths of multiple optimization strategies, they effectively balance trade-offs between coverage, connectivity, energy efficiency, and latency. Their flexibility and optimization power make them particularly well-suited for complex and dynamic environments such as disaster zones or smart cities. Hybrid algorithms enhance solution quality, robustness, and adaptability, maintaining consistent performance even under high mobility and network variability. They are also effective in improving Quality of Service (QoS) metrics such as throughput, packet delivery ratio, and link stability, especially when combining global search with local refinement methods.

However, to fully enable real-time deployment, further research is needed to reduce execution time and improve responsiveness. To further advance their applicability, future research could explore lightweight hybridization strategies, hardware acceleration techniques (e.g., GPU or FPGA-based computation), and the integration of learning-based components for enhanced adaptability in highly dynamic and large-scale UAV networks. Designing modular hybrid frameworks that can be dynamically reconfigured based on network conditions would improve flexibility and operational efficiency in practical deployments.

Moreover, future hybrid algorithms could be developed for UAV networks and low-altitude systems [109], incorporating emerging technologies such as intelligent reflecting surfaces (IRSs) [110], rate splitting multiple access (RSMA), and integrated sensing and communication (ISAC) [111] to enhance network performance in terms of capacity, reliability, and spectral efficiency. In addition to these physical- and medium-access-layer innovations, there is increasing interest in leveraging large AI models to support autonomous decision-making, adaptive resource allocation, and real-time network optimization in UAV-assisted systems. Large AI models, particularly those trained on extensive communication, mobility, and environmental datasets, can enable UAV networks to dynamically predict channel states, optimize node placement, adapt trajectories, and manage interference in complex and dense deployment scenarios. Such models can also facilitate context-aware beamforming, intelligent traffic offloading, and proactive fault prediction, advancing beyond traditional rule-based or shallow learning approaches.

Recent research [112] suggests that embedding large AI models within UAV networks can support a range of intelligent applications, including real-time situational awareness, coordinated multi-UAV control, and adaptive mission planning, which are critical for emerging use cases like disaster response, smart cities, and low-altitude economy ecosystems. These models can operate either on board high-capability UAVs or in distributed edge/cloud frameworks, balancing local responsiveness with global knowledge aggregation. Integrating AI capabilities with IRS, RSMA, and ISAC can further enhance network adaptability by enabling joint optimization across propagation environment shaping, multiple access strategies, and sensing-aided communication, leading to more resilient and efficient UAV communication systems.

Exploring large-AI-model-enabled UAV networks represents a promising research direction that complements hybrid algorithm development and emerging technologies, offering deeper insights into autonomous, intelligent, and adaptive low-altitude communication infrastructures.

Table 1 summarizes key studies that address UAV node placement problem, highlighting the distinguishing features of each algorithm along with the type of UAV communication network considered, the evaluation metrics, and the simulation platforms.

Table 2 presents an overview of selected meta-heuristic algorithms for UAVCN node placement optimization. It summarizes key studies that address the node placement problem of UAVCN, highlighting the performance summary of each.

**Figure 19:**
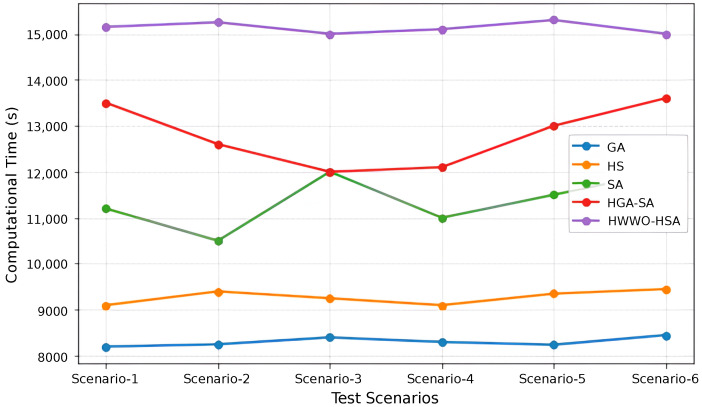
Computational time over various scenarious.

**Figure 20:**
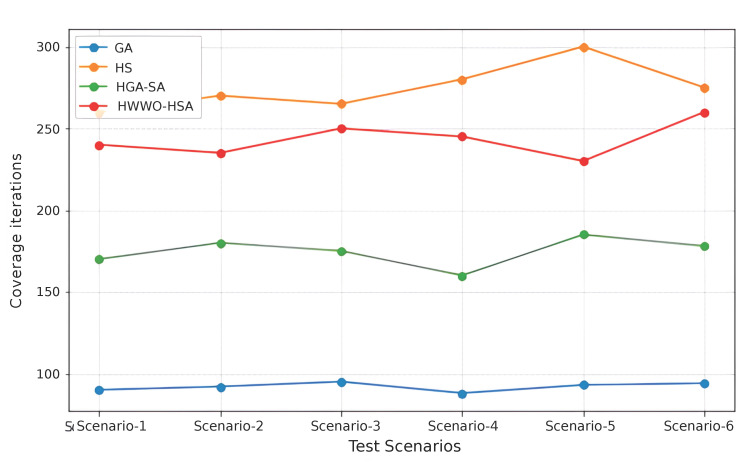
Coverage over various scenarious.

While Table 1 and Table 2 provide a comprehensive overview of existing methods, the experimental evaluation uses the dataset described in [20] to compare a selected set of representative algorithms based on computational time and coverage, as illustrated in Figure 19 and Figure 20. The algorithms were executed in the Python environment using the publicly available implementations provided in [20], ensuring a uniform and reproducible computational framework. The analysis focuses on small- and medium-scale scenarios. Specifically, 6 test scenarios were considered: Scenario 1 with 10 nodes, Scenario 2 with 20 nodes, Scenario 3 with 30 nodes, Scenario 4 with 40 nodes, Scenario 5 with 50 nodes, and Scenario 6 with 60 nodes.

Figure 19 illustrates the computational time of five meta-heuristic algorithms, GA, HS, SA, HGA-SA, and HWWO-HSA, evaluated on a wireless mesh topology across six test scenarios. The results show that GA and HS consistently exhibit the lowest execution times, ranging approximately from 8100 to 9400 s, indicating that both algorithms require relatively modest computational resources and maintain stable performance as the network size increases. The SA algorithm demonstrates moderate computational cost, with execution times varying between roughly 10,500 and 12,000 s across the evaluated scenarios. In comparison, HGA-SA presents higher execution times, ranging from approximately 12,000 to 13,500 s, which can be attributed to the additional processing introduced by hybridization and multi-stage search operations. The HWWO-HSA algorithm exhibits the highest computational time among the evaluated methods, with execution times between roughly 15,000 and 15,500 s across all scenarios. While this approach achieves strong optimization performance, it also incurs increased computational overhead, likely associated with its multi-phase search structure and more complex update mechanisms.

Figure 20 illustrates the coverage performance of four meta-heuristic algorithms GA, HS, HGA-SA, and HWWO-HSA evaluated across six identical mesh-network test scenarios. Overall, the results reveal clear performance differences among the algorithms, highlighting variations in exploration capability and solution quality. HS consistently achieves the highest coverage across all scenarios, followed closely by HWWO-HSA, indicating that algorithms with strong or hybridized exploration mechanisms are more effective in identifying well-distributed UAV placements. HGA-SA demonstrates moderate coverage performance, reflecting a balance between local refinement and global search, albeit with slightly lower coverage compared to HS and HWWO-HSA. GA exhibits substantially lower coverage across all scenarios, suggesting limited exploration capability and reduced effectiveness in handling increasing network sizes under the considered conditions.

These results collectively indicate that enhanced exploration strategies and hybrid search designs play a critical role in improving coverage performance in mesh-based UAV deployment scenarios.

## 6. Conclusions

This comprehensive review underscores the pivotal role of MHAs in addressing the UAV node placement problem within UAVCNs, a task recognized as NP-hard due to its multidimensional optimization nature. UAVCNs, through their dynamic, scalable, and infrastructure-independent architectures, have introduced new possibilities for maintaining connectivity in constrained or rapidly changing environments. However, the challenge of determining optimal node deployment remains central, with direct implications for coverage quality, link reliability, network throughput, latency, and energy consumption.

Metaheuristic approaches ranging from evolutionary algorithms and swarm intelligence to physics-based and hybrid models have shown themselves to be practical tools for handling the complexity of this problem. Their strength lies in flexibility, the ability to adapt to changing conditions, and their competence in finding near-optimal solutions where deterministic methods may become computationally infeasible. In many applications, these algorithms are favored for their capacity to explore diverse solutions and adjust to different deployment scenarios, including urban layouts, rural terrains, and disaster zones.

The selection of an appropriate algorithm typically depends on the problem’s operational context. Factors such as mission goals, environmental dynamics, platform constraints, and performance priorities play a significant role in determining algorithm suitability. For instance, certain algorithms may perform better in sparse environments where wide coverage is key, while others may be more efficient in dense scenarios requiring careful load balancing and minimal energy consumption.

An increasing interest in hybrid and adaptive algorithms suggests a shift toward more context-aware optimization strategies. These methods aim to combine the strengths of multiple meta-heuristics such as global exploration with local refinement and sometimes integrate learning components to respond to user mobility or topological changes. While these developments offer potential benefits, challenges remain, especially in balancing conflicting objectives like energy efficiency and latency or ensuring real-time responsiveness under uncertain conditions.

Future work may focus on enhancing these methods with lightweight learning modules, cross-layer awareness, real-time feedback, and hybrid optimization strategies to better support emerging applications in smart cities, emergency response, and beyond. In addition, the integration of MHAs with edge computing paradigms, 6G-enabled UAV systems, and distributed intelligence frameworks can provide a foundation for ultra-reliable and low-latency decision-making. The development of hybrid MHA formulations that combine complementary search behaviors is expected to be particularly valuable in addressing the complexity and dynamic nature of UAV communication networks. Developing standardized benchmarks and reproducible evaluation frameworks will also be critical for the fair assessment and advancement of MHA-based solutions in practical UAVCN deployments.

## Figures and Tables

**Table 1:** Features of meta-heuristic algorithms (MHAs).

MHA Type	Ref./Method/Scenario	Metrics	Features	Platform
**Evolutionary Algorithms**	[79]/GA/UAV-Based Relay Networks	Throughput	By using K-means to seed the initial UAV positions, the GA-based allocation shortens convergence time, maintains manageable complexity and fairness, and ultimately delivers higher total throughput in the HAP–UAV integrated network.	Matlab 9.12 R2022a
	[81]/GA, SA/UAV-assisted Cellular Network	Coverage, delay, throughput	By modeling drone-BS placement as a linear optimization problem and employing both GA and SA, the study determines the optimal number and positions of drones for 5G coverage, minimizing cost while satisfying coverage, data-rate, latency, and throughput constraints.	Java, Python
	[52]/GA/FANET	Throughput	By dynamically repositioning the UAVs and analysing how the Radius of Position Constraint (RPC) and Radius of Particle Size (RPS) influences feasible movements, the GA approach successfully maximizes network throughput.	Matlab
	[80]/GA, HCA/MANET	Connectivity	By deploying UAVs in disaster scenarios, the integration of Genetic Algorithms and Hill Climbing optimization enhances communication efficiency by optimally positioning UAVs to maximize coverage for ground users.	Matlab
	[67]/GA/UAV Swarm Networks	Coverage, energy consumption	By integrating a bi-layer optimization procedure with energy-aware constraints, the GA enables optimal planning of UAV quantity and placement to ensure seamless and persistent surveillance coverage around a ground vehicle in drone-truck search-and-rescue operations.	Matlab
	[68]/GA + heuristic/UAV-Based Relay Networks	Coverage	By designing an efficient strategy for UAV relay node placement, the combination of the smallest enclosing circle method with a GA minimizes the number of required UAVs while ensuring reliable connectivity for all ground terminals.	Matlab
	[96]/GA + heuristic/UAV-Based Relay Networks	PDR	By determining the optimal positioning of UAVs, NSGA-II addresses a multi-objective optimization problem by generating Pareto-optimal solutions that balance the minimization of UAV count with the maximization of PDR.	C++
	[16]/MOPSO, NSGA-II, SPEA2, PESA II/AMN	Coverage, QoS, energy consumption	By analyzing altitude-aware UAV placement in post-disaster mesh networks with a focus on optimizing coverage, QoS, and energy efficiency, four meta-heuristics were evaluated based on performance metrics including generational distance, diversification, spread, and domination across varying scenario scales.	Matlab, ns-2.35
	[78]/MLMPGA (Multi-layout multi-subpopulation genetic algorithm)/AMN	Coverage	By addressing the NP-hard problem of UAV-network deployment through a multi-objective optimization framework, MLMPGA optimizes coverage, fault-tolerance, and redundancy by using multiple evolving subpopulations with different layouts to enhance search diversity and solution quality.	Python 2.7
	[21]/DNSGA (Directed-Evolution Non-Dominated Sorting GA)/FANET	Latency, load imbalance	By leveraging a directed-evolution non-dominated sorting genetic algorithm (DNSGA) with a K-means-based task assignment and efficiency-driven load migration strategy, the approach efficiently optimizes edge server placement in UAV ad hoc networks, jointly minimizing worst-case transmission latency and load imbalance while ensuring fast convergence, diverse high-quality solutions, and adaptability across different network scales.	Python/ Matlab
	[54]/DEVIPS/ILN	Energy consumption, number of stop points	By formulating UAV deployment for IoT data collection as a variable-length optimization problem, DEVIPS was introduced to adaptively determine both the number and locations of UAV stop points using evolutionary operations tailored to dynamic solution structures.	Matlab
	[22]/DEA, CUCO, HBA/FANET	Throughput, PDR, and delay	By comparing CUCO, DEA, and HBA for optimizing contention window size, a Markov chain-based model was used to establish parameter relationships and derive key performance metrics such as throughput, delay, and PDR.	Matlab
	[83]/DE/UAV assisted Cellular Network	Coverage	By constructing a multi-UAV-enabled MEC system, a DE-based deployment mechanism was proposed to optimize UAV positioning for load balancing, coverage, and QoS assurance, alongside a deep reinforcement learning algorithm for efficient task scheduling within individual UAVs.	Matlab
**Nature inspired algorithms**	[95]/IAWOA/UAV assisted Cellular Network	Accuracy, throughput	By formulating the 3D UAV placement problem in an IIoT network with NOMA support, an Improved Adaptive Whale Optimization Algorithm (IAWOA) is employed for offline optimization, and a Path Aggregation Network (PANet) was introduced to enable efficient real-time UAV deployment.	Python
	[55]/PSO-L/UAV-Based Relay Networks	Energy efficiency	By jointly designing hybrid precoding and UAV positioning in a mmWave MU-MIMO system, SVD- based RF beamforming, RZF precoding, and PSO-based UAV placement were utilized to enhance spectral and energy efficiency.	Matlab
	[85]/PSO/UAV swarm networks	Accuracy and convergence distance	By incorporating height-aware sensing into a PSO-based multi-source localization framework, UAVs were enabled to dynamically adjust their 3D positions for efficient source detection, balancing wide-area coverage at high altitudes with precise localization at lower altitudes.	Matlab R2019a
	[87]/PSO/FANET	Percentage of victims discovered, time to discover victims, connectivity	By leveraging a PSO-based algorithm (dPSO-U) integrated with Delay Tolerant Networking, UAVs are enabled to dynamically explore disaster scenarios and autonomously converge toward victim clusters, offering faster victim discovery, improved connectivity, and optimized parameter configurations compared to traditional trajectory planning methods.	Matlab
	[72]/PSO and KTS/UAV assisted Cellular Network	Coverage density	By integrating PSO and K-means with Ternary Search (KTS) for single UAV 3D placement, and Circle Packing Theory (CPT) with altitude optimization for multi-UAV deployment, energy-efficient UAV positioning was achieved to maximize coverage density across various region shapes.	Matlab
	[88]/PSO/UAV assisted Cellular Network	Coverage	By utilizing PSO and the Hata-Okumura path loss model, UAV base station deployment in open areas was evaluated, demonstrating how UAV coverage was influenced by antenna range and quantity.	Matlab
	[65]/PSO/UAV assisted Cellular Network	Throughput, SNR	By employing PSO for dynamic 3D UAV placement, drone-mounted LTE base station positioning and resource allocation were optimized to maximize coverage while meeting diverse QoS requirements with reduced computational complexity.	Matlab
	[70]/PSO/UAV-SDN-Enabled Networks	Coverage, latency and packet loss	By integrating SDN, the spring virtual force method, and an improved PSO algorithm, manageable topology formation in FANETs was achieved to ensure safe spacing, adequate link quality, wide area coverage, and seamless end-user mobility with reliable network connectivity.	Python, C++ and OMNeT++
	[77]/PSO/UAV assisted Cellular Network	Coverage, voice quality and user density	By addressing the 3D drone placement problem using PSO, a hierarchical UAV architecture with access and distribution drones was introduced to efficiently deliver VoWiFi service, minimizing the number of drones while ensuring sufficient coverage and voice quality across varying terrain sizes and user densities.	Matlab 2020a
	[59]/PSO and EML/UAV assisted Cellular Network	Coverage	By applying PSO- and EML-based algorithms, the 3D placement of multiple drone base stations was optimized to maximize coverage and ensure efficient deployment for both uniform and non-uniform user distributions.	Matlab
	[76]/PSO, K-means algorithm, GA and ABS/UAV assisted Cellular Network	Coverage	By combining PSO-based clustering with PSO-based 3D UAV placement, the number of UAVs and transmit power were minimized while ensuring full user coverage and significantly reducing execution time.	Matlab
	[71]/PSO and K-means/UAV assisted Cellular Network	Packet loss, latency, coverage	By integrating an improved PSO algorithm with K-means clustering, the number and 3D placement of drone base stations were jointly optimized to restore coverage in disaster scenarios.	Mininet-Wifi
	[18]/SBA/ILN	Coverage	By leveraging a parallelized SBA, the proposed approach enables efficient UAV placement by effectively handling nonlinear, mixed, and multimodal optimization problems, while also employing a range of test suites that vary in size from small to large to ensure adaptability and robustness across diverse deployment scenarios.	Python 3.8
	[60]/GWO/UAV assisted Cellular Network	Coverage	By applying stochastic geometry for SINR-based downlink coverage evaluation and employing the Grey Wolf Optimizer, optimal Drone-BS placement in 5G networks was achieved to enhance user coverage in dense urban environments in line with 3GPP objectives.	Matlab
	[58]/SSO/UAV assisted Cellular Network	Coverage	By modeling a realistic constrained scenario with 3GPP-compliant channel characterization, including backhaul and interference constraints, a scalable Social Spider Optimization (SSO) algorithm was introduced to optimize UAV placement and association with user equipment and ground base stations for enhanced network coverage.	Matlab
	[57]/EHO/UAV assisted Cellular Network	Coverage	By adopting the Elephant Herding Optimization (EHO) algorithm, the static drone location problem was addressed by minimizing the number of deployed drones while ensuring full target coverage and enabling efficient monitoring in both uniform and clustered target distributions.	Visual Studio 2017
	[19]/MPA/UAV assisted Cellular Network	Coverage	By enhancing the Marine Predators Algorithm with chaotic maps and opposition-based learning, the NP-hard problem of UAV-BS placement was addressed by optimizing drone positions and altitudes in static deployment scenarios.	Matlab
	[61]/SA/UAV assisted Cellular Network	QoS, throughput	By formulating a coverage-maximization problem and applying a SA algorithm, a dynamic UAV-BS placement strategy was introduced to enhance communication coverage while ensuring collision avoidance among UAVs.	Matlab
	[64]/DA/ILN	Coverage, connectivity	By formulating the UAV relay placement task as a clustering problem with a summation-form distortion function, the Deterministic Annealing (DA) algorithm was applied to determine the minimal number and optimal locations of UAVs, ensuring full and reliable network connectivity while maintaining scalability and avoiding local minima.	Matlab
	[73]/SA/UAV assisted Cellular Network	Energy efficiency	By jointly optimizing power allocation and 3D UAV placement using fractional programming and SA, energy efficiency in FD-NOMA URLLC systems under finite blocklength was enhanced.	Matlab
**Local Search Algorithms**	[66]/LSAO/MANET	Coverage, connectivity, energy consumption, load distribution	By modeling UAV placement as a constraint-based optimization problem, the LSAO algorithm was proposed for efficient deployment in MANETs, achieving superior performance and validated on standard placement test cases.	Matlab R2021
	[99]/TS/SDN	Throughput	By leveraging traffic-aware A2A link demands and flow paths, UAV positions were dynamically determined using a centralized TS-based approximation algorithm executed on an SDN controller to maximize overall system throughput through demand-driven placement.	Matlab
**Hybrid Algorithms**	[74]/Hybrid SA–greedy algorithm (HSA-G)/UAV assisted Cellular Network	Overall outage probability (OOP), individual outage probability (IOP), energy consumption	By integrating a non-cooperative game model and hybrid SA-greedy algorithms, power allocation and UAV access point placement were jointly optimized to counteract jamming attacks, minimize outage probabilities, and enhance communication reliability in uplink NOMA systems over Nakagami-m fading channels.	Matlab
	[93]/Hybrid PSO with SA (HPSO)/UAV-Based Relay Networks	Minimum achievable rate (worst-user rate)	By jointly optimizing UAV placement and beam-forming in mmWave multicast systems using HPSO and BCD algorithms, user cluster rates were enhanced under building blockages with the aid of UAV-mounted intelligent reflecting surfaces (IRSs).	Matlab
	[56]/Hybrid MHA (PSO and Hill Climbing)/IBN	Sum rate	By decomposing the joint UAV placement and RB allocation into a two-layer approach, hill-climbing was used for RB assignment and PSO for UAV positioning to maximize uplink sum rate in a NOMA-enabled environment. The method considers user fairness, dynamic channels, UAV altitude, position, and power constraints, offering modularity and scalability for multi-user uplink scenarios.	Matlab
	[46]/Hybrid MHA/UAV-Based Relay Networks	Connectivity, link capacity	By modeling UAV relay placement as a single allocation p-hub median problem and applying a hybrid MHA, A2G and A2A link capacities were optimized while ensuring full user connectivity with minimal computation time.	Java R 7SE, ILOG CPLEX Version 12.10.0 by IBM
	[20]/HWWO-HSA and HGA-SA/AMN	SNR, relative percentage deviation (RPD), Computational time, Average computation time and Coverage iterations	By formulating the UAV placement problem in 3D space, two hybrid meta-heuristic algorithms HGA-SA and HWWO-HSA are proposed that combine graph-based connected component analysis and Taguchi-tuned parameters to preserve network connectivity and improve solution quality in terms of coverage, while incurring higher computational time than non-hybrid methods, reflecting the trade-off observed between coverage performance and computational cost.	Matlab, ns-2.35, Minitab
	[15]/IMRFO-TS/AMN	Coverage, connectivity, energy consumption, and load distribution	By hybridizing the IMRFO algorithm with TS and incorporating a tangential control strategy, the IMRFO-TS algorithm was proposed to solve the UAV placement problem in smart cities, demonstrating its effectiveness across 52 benchmark scenarios.	Matlab R2021b

**Table 2:** Comparative performance of MHAs for UAV placement optimization.

Alg./Ref.	Compared Algorithms	Performance Summary
SBA/[18]	GA, PSO, RW	SBA outperforms GA, PSO, and RW in 11 of 12 problems, achieving up to 33.33% fewer UAVs (from 120 to 80), 80.6% faster execution time (202.4 s → 39.23 s), and superior coverage across sensor sets: 6/8 (20 sensors), 32/42 (100), 83/113 (200), and 153/224 (500).
CUCO, HBA, DEA/[22]	IEEE 802.11 MAC (CSMA/CA, RTS/CTS)	Under RTS/CTS, CUCO achieves up to 6.97% higher throughput (5.7520 vs. 5.3775 at 10 drones). Under CSMA/CA, it achieves up to 5.67% improvement (7.4810 vs. 7.0797 at 10 drones) and 3.81% at 50 drones (7.2746 vs. 7.0073).
GA-based algorithm/[79]	Exhaustive Search, PSO	Achieves up to 32% higher throughput than HAP-only at 100 m. Associates 91 users (<50%) with UAVs. Performs best when radius > 100 m. GA and PSO show similar throughput (∼6.5–7.2 × 10^7^ bps), both faster than exhaustive search.
SA, GA/[81]	SA and GA	SA is faster in small areas (≤44 km^2^), while GA performs better for large-scale scenarios with more stable convergence. Both yield identical UAV placements. As UAV height increases (10–50 m), energy consumption increases, packet delay drops (0.032 s → 0.015 s), and throughput increases up to 5.5 Mbps.
DE + GA (Bi-layer)/[67]	GA	Achieves seamless coverage using only 3 UAVs with 19.60 Wh total overhead, compared to 5–9 UAVs and up to 34.55 Wh for GA-only. Improves energy efficiency, precision, and handles multiple constraints more effectively than GA alone.
LSAO/[66]	FA, SCA, GWO, MRFO, IMRFOTS, AO	Outperforms all algorithms in fitness, coverage, and connectivity for 4 test cases and 3 UAV counts (5, 10, 15). Achieves 100% coverage in 7 of 12 scenarios, maximum fitness 42.51 (Case I, 10 UAVs), and minimum standard deviation (0.65). Statistically superior in 10/12 Wilcoxon tests (*p* < 0.05).
CSBWOA/[84]	BPSO, K-means, GWO	Shows best results in energy, cluster lifetime, and node survival for densities 20–110. At 35 UAVs: lowest mean energy (2.4 J), cluster time 1.8 s (better than BPSO), longest cluster lifetime (410 rounds), and highest survival rate (80%).
HGA-SA, HWWO-HSA/[20]	GA, HS, SA, WWO	HGA-SA demonstrates strong performance in small-scale problem settings (10–40 targets), achieving approximately 11–12% improvement in fitness compared to GA and HS. In contrast, HWWO-HSA attains higher solution quality in medium- and larger-scale scenarios (50–120 targets), with performance gains of up to 23.2% relative to WWO. While both hybrid algorithms tend to converge in fewer iterations, this improvement is accompanied by increased computational cost. In particular, HGA-SA incurs higher runtime compared to simpler evolutionary and swarm-based methods, and HWWO-HSA exhibits the largest computational overhead, with computational time approximately 18.4% higher than that of standard population-based algorithms. These observations are consistent with the computational time and coverage trends reported in Figure 19 and Figure 20, where hybrid methods achieve improved solution quality at the expense of increased runtime.
IMRFO-TS/[15]	TS, BA, FA, GWO, SCA, WOA, MRFO, RSA	Achieves best fitness in all 52 benchmarks: up to 87% improvement vs. RSA and 47% vs. MRFO. Maintains >99% connectivity and up to 99.88% coverage. Reduces energy by up to 31.85%. Lowest load distribution (5.54 vs. 29.74 in RSA). Supports higher density with fewer UAVs and stable convergence.

## Data Availability

Data are contained within the article.
